# Age-Dependent Defects of Regulatory B Cells in Wiskott-Aldrich Syndrome Gene Knockout Mice

**DOI:** 10.1371/journal.pone.0139729

**Published:** 2015-10-08

**Authors:** Tadafumi Yokoyama, Ayumi Yoshizaki, Karen L. Simon, Martha R. Kirby, Stacie M. Anderson, Fabio Candotti

**Affiliations:** 1 Genetics and Molecular Biology Branch, National Human Genome Research Institute, National Institutes of Health, Bethesda, Maryland, United States of America; 2 Medical Genetics Branch, National Human Genome Research Institute, National Institutes of Health, Bethesda, Maryland, United States of America; 3 Department of Dermatology, Faculty of Medicine, University of Tokyo, Tokyo, Japan; 4 Division of Immunology and Allergy, University Hospital of Lausanne, Lausanne, Switzerland; INSERM-Université Paris-Sud, FRANCE

## Abstract

The Wiskott-Aldrich syndrome (WAS) is a rare X-linked primary immunodeficiency characterized by recurrent infections, thrombocytopenia, eczema, and high incidence of malignancy and autoimmunity. The cellular mechanisms underlying autoimmune complications in WAS have been extensively studied; however, they remain incompletely defined. We investigated the characteristics of IL-10-producing CD19^+^CD1d^high^CD5^+^ B cells (CD1d^high^CD5^+^ Breg) obtained from *Was* gene knockout (WKO) mice and found that their numbers were significantly lower in these mice compared to wild type (WT) controls. Moreover, we found a significant age-dependent reduction of the percentage of IL-10-expressing cells in WKO CD1d^high^CD5^+^ Breg cells as compared to age-matched WT control mice. CD1d^high^CD5^+^ Breg cells from older WKO mice did not suppress the *in vitro* production of inflammatory cytokines from activated CD4^+^ T cells. Interestingly, CD1d^high^CD5^+^ Breg cells from older WKO mice displayed a basal activated phenotype which may prevent normal cellular responses, among which is the expression of IL-10. These defects may contribute to the susceptibility to autoimmunity with age in patients with WAS.

## Introduction

The Wiskott-Aldrich syndrome (WAS) is a rare X-linked primary immunodeficiency that causes recurrent infections, thrombocytopenia, eczema, and high incidence of malignancy in affected patients. Autoimmune complications are also common in WAS and have been described in 40% to 70% of patients in retrospective cohort studies [1, 2].

WAS is caused by mutations of the *WAS* gene that encodes for the WAS protein (WASp), a multidomain-containing protein that regulates the actin cytoskeleton in hematopoietic cells [3]. The role of WASp deficiency in the development of autoimmunity in WAS has been explored extensively. Others and we have demonstrated that WASp-deficient natural regulatory T cells (nTreg) have defective suppression function of effector T cells and of B lymphocyte proliferation [4, 5, 6, 7, 8]. Recent observations in WASp-deficient (WKO) mice have shown decreased numbers of interleukin (IL)-10-producing regulatory B cells (Breg), associated with increased Th1 and Th17 cells, as well as exacerbated pathology in an experimental mouse arthritis model [9,10]. Altogether, these findings indicate the importance of WASp at multiple levels of the regulatory cellular network of the immune system. We and others have also shown that both humans affected with WAS and mouse models of the disease present with intrinsic defects of B cell differentiation that are associated with increased production of autoantibodies [11, 12, 13]. In addition, we have observed that WASp deficiency results in age-dependent attrition of the immune system in humans [13, 14]. These clinical observations are reflected in the WKO mouse model where we observed that animals older than 6 months of age show significantly higher titers of antinuclear (ANA) and anti dsDNA antibodies and increased severity of proliferative glomerulonephritis compared to age-matched wild type (WT) controls [15, 16].

Based on these findings we set out to assess the presence, function, and effects of age on splenic CD1d^high^CD5^+^ B cells (CD1d^high^CD5^+^ Breg), a subset of regulatory B cells that have potent immune inhibitory capabilities through the secretion of IL-10 [17, 18, 19]. CD1d^high^CD5^+^ Breg cells affect CD4^+^ T cell production of interferon- (IFN-) and tumor necrosis factor- (TNF-) and are thought to thereby regulate the differentiation of CD4^+^ T cells into Th1/Th2/Th17 cells. CD1d^high^CD5^+^ Breg cells are also known to play a critical role in the pathogenesis of various autoimmune mouse models, such as experimental autoimmune encephalomyelitis (EAE) and murine systemic lupus erythematosus [18, 19].

However, while it has been reported that WKO mice have lower numbers of IL-10-producing, B220^+^ regulatory B cells [9, 10], detailed information about the specific CD1d^high^CD5^+^ subset of Breg cell in WKO mice is lacking. In this study, we demonstrate that CD1d^high^CD5^+^ Breg cells are reduced in numbers in WKO mice and become functionally impaired in older animals, which provides additional insights into the mechanisms leading to immune dysregulation in WAS.

## Materials and Methods

### Mice

*Was* ^-/y^, *Was* ^-/-^ (129S6/SvEvTac-Was^tm1Sbs^/J) (WKO), and 129S6/SvEvTac (WT) mice were used in experiments approved by the National Human Genome Research Institute Animal Care and Use Committee. All mice were bred in a specific pathogen-free barrier facility and used from 6 weeks to under 1.5 years of age.

### Cell preparations

Single-cell suspensions from spleens were generated by gentle dissection and treatment in ACK lysis buffer (Lonza, Walkersville, MD, USA) followed by passage through 70 μm cell strainers (BD Biosciences, San Jose, CA, USA) and suspension in complete medium (RPMI 1640 media containing 10% FCS, 200 μg/ml penicillin, 200 U/ml streptomycin, 4 mM L-glutamine and 5×10^−5^ M 2-mercaptoethanol (all from Gibco, Gaithersburg, MD, USA).

A MACS cell separator (Milteny Biotech, Auburn, CA, USA) was used to purify lymphocyte populations according to the manufacturer’s instructions. CD4^+^ T cells were positively selected from splenocytes using anti-mouse CD4-coated microbeads (Milteny Biotech). The cells were enriched a second time using a fresh MACS column to obtain >95% cell purities.

To purify CD19^+^CD1d^high^CD5^+^ Breg cells, CD4-depleted cells were further subjected to cell sorting using a BD FACSAria III sorter (BD Biosciences) based on their expression of CD19, CD1d, and CD5 with obtained purities of >95%.

### Flow cytometry analysis of cell surface markers and intracellular IL-10 synthesis

FITC-, PE-, APC-, PerCP-, or APC-Cy7-conjugated anti-CD19 (1D3), CD5 (53–7.3), CD1d (1B1), CD4 (RM4-5), CD45R/B220 (RA3-6B2), CD69 (H1.2F3), IL-10 monoclonal antibodies and isotype controls were obtained from BD Biosciences and Biolegend, San Diego, CA, USA. Staining of cell surface markers was performed using standard procedures. The gating strategy used to assess the frequency of CD1d^high^CD5^+^ Breg cells in mouse spleens are described in Figure in S1 Fig. For intracellular staining of IL-10 production, isolated splenocytes were resuspended (2×10^6^ cells/ml) in medium containing LPI (10 μg/ml lipopolysaccharides (LPS; Sigma Aldrich, St. Louis, MO, USA), 50 ng/ml phorbol 12-myristate 13-acetate (PMA; Sigma Aldrich), 500 ng/ml ionomycin (Sigma Aldrich)), and 2 μl of GolgiStop (BD Biosciences) and incubated with 5% CO_2_ at 37°C for 5 hrs (LPI stimulation). Fc receptors were then blocked with purified rat anti-mouse Fc-receptor-specific mAb (CD16/CD32 (2.4G2) antibody; BD Biosciences) before cell-surface staining and then fixed and permeabilized with the Cytofix/Cytoperm kit (BD Biosciences) according to the manufacturer’s instructions. Background staining was assessed using non-reactive, isotype-matched control mAbs. We defined IL-10-positive cells by gating to exclude >99.9% of non-reactive cells. All data were collected on FACS Calibur and LSRII instruments (BD Biosciences) and analyzed with FlowJo software (TreeStar, Ashland, OR, USA).

### ELISA

Purified CD1d^high^CD5^+^ Breg cells (1×10^5^ cells/ml) were cultured in 1 ml of complete medium with or without LPI for 5 hrs. IL-10 concentration in culture supernatant was then quantified using the IL-10 OptEIA ELISA kit (BD Pharmingen, San Diego, CA, USA) following the manufacturer’s protocol. All assays were conducted in triplicate.

### Cell co-culturing assay

Isolated WT CD4^+^ T cells and WT/WKO CD1d^high^CD5^+^ Breg cells were cultured in complete medium in 96-well flat-bottom plates in the presence of plate-bound anti-CD3 (0.1 μg) and soluble anti-CD28 (1 μg/ml) antibodies (eBioscience, San Diego, CA, USA). For co-culture experiments, WT CD4^+^ T cells (1×10^5^ cells in 100μl/well) and either WT or WKO CD1d^high^CD5^+^ Breg cells were cultured at a ratio of 1:1 for 72 hrs.

After stimulation, concentration of cytokines in culture supernatants was measured using the IFN- ELISA MAX^TM^ kit (Biolegend), and the mouse-TNF- kit (OptEIA ELISA; BD Pharmingen) following the manufacturer’s protocols.

Cultured cells were stained with anti-CD45R/B220, CD19 and CD69. CD4^+^ T cells were analyzed as CD19-negative, B220-negative cells. Dead cells were excluded using propidium iodide staining. All experiments were repeated at least three times. For all experiments, age-matched WT control mice were used. Pooling of splenocytes from multiple mice was used, if necessary, for examination of minor cell subsets.

### Statistical analysis

Data are shown as mean and standard deviation (SD). Significant differences (p<0.05) between sample means were determined using Student’s *t*-test. Percentage values were statistically analyzed after angular transformation and using the Mann-Whitney test.

## Results and Discussion

The number of splenocytes obtained from WKO mice (8.5±3.68×10^7^ cells/mouse; N = 45) was not significantly different from that of WT animals (9.4±3.64×10^7^ cells/mouse; N = 76). However, and as expected (3, 9, 10), the number of total splenic CD19^+^ B cells observed in WKO mice (2.6±1.23×10^7^ cells/mouse; N = 44) was significantly lower than in WT mice (3.7±1.83×10^7^ cells/mouse; N = 75) (p<0.05).

**Fig 1:**
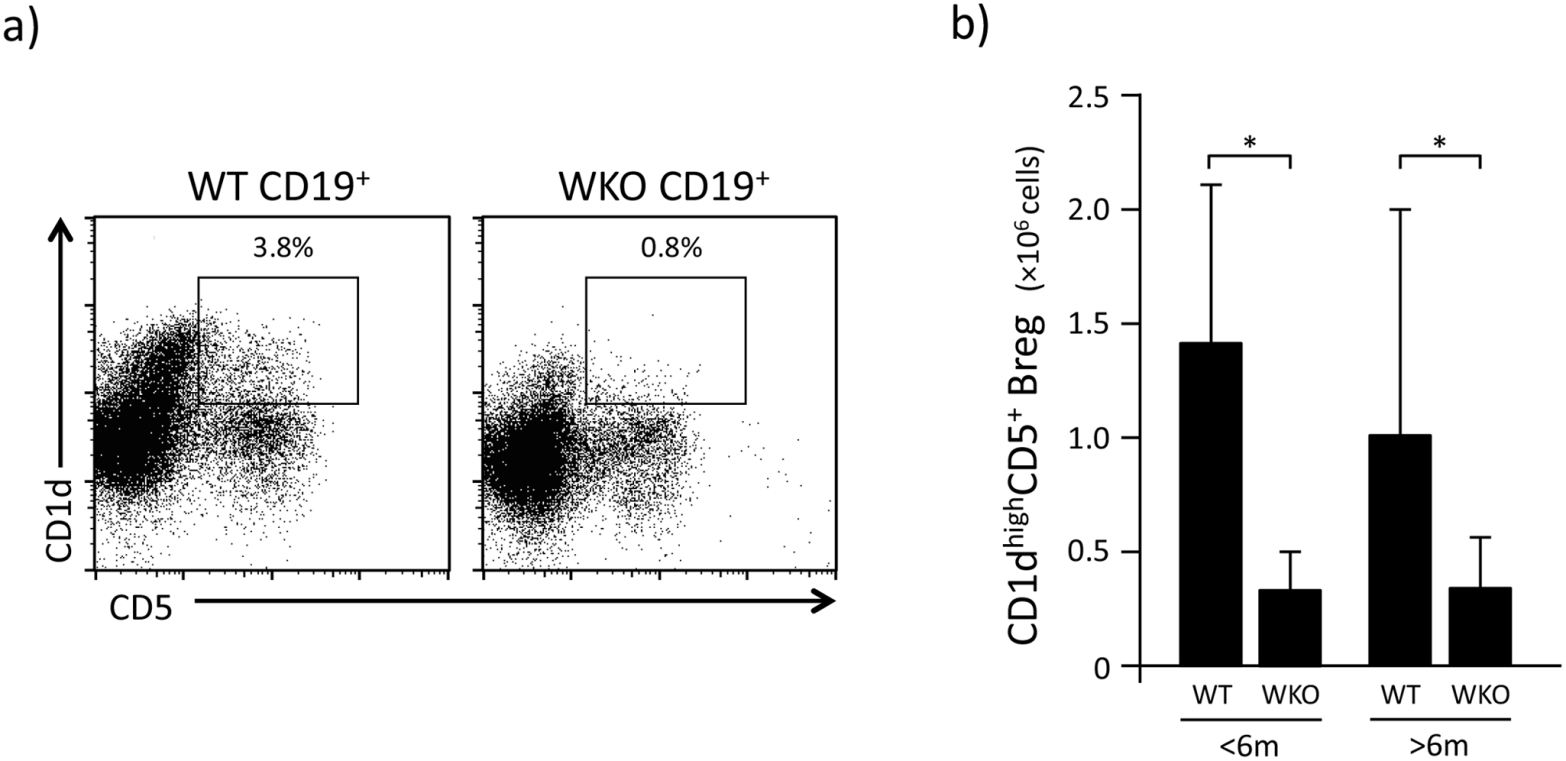
Characteristics of CD1d^high^CD5^+^ Breg cells in WKO mice. a: Representative results of the percentages of CD1d^high^CD5^+^ Breg cells detected within total splenic CD19^+^ B cells of WT and WKO mice. b: Total number of splenic CD1d^high^CD5^+^ Breg cells in younger (<6 months old) and older (>6 months old) mice. Average numbers (±SD) of CD1d^high^CD5^+^ Breg cells from 23–49 mice are shown. *p<0.002 (Student’s *t*-test).

The percentage of CD1d^high^CD5^+^ Breg cells within WKO splenic CD19^+^ B cells was statistically lower than in WT splenic B cells (1.3±0.75% vs 3.8±2.89%, p<0.05) (Fig 1a). Consequently, the absolute number of CD1d^high^CD5^+^ Breg cells in WKO mice (0.3±0.22×10^6^ cells/mouse) was approximately 5 times lower than in WT animals (1.5±1.75×10^6^ cells/mouse, p<0.05). Age did not appear to be a factor in this observation, as both younger (<6 months of age) and older (>6 months of age) WKO mice presented significantly lower number of CD1d^high^CD5^+^ Breg cells compared to WT controls (Fig 1b, p<0.05).

**Fig 2:**
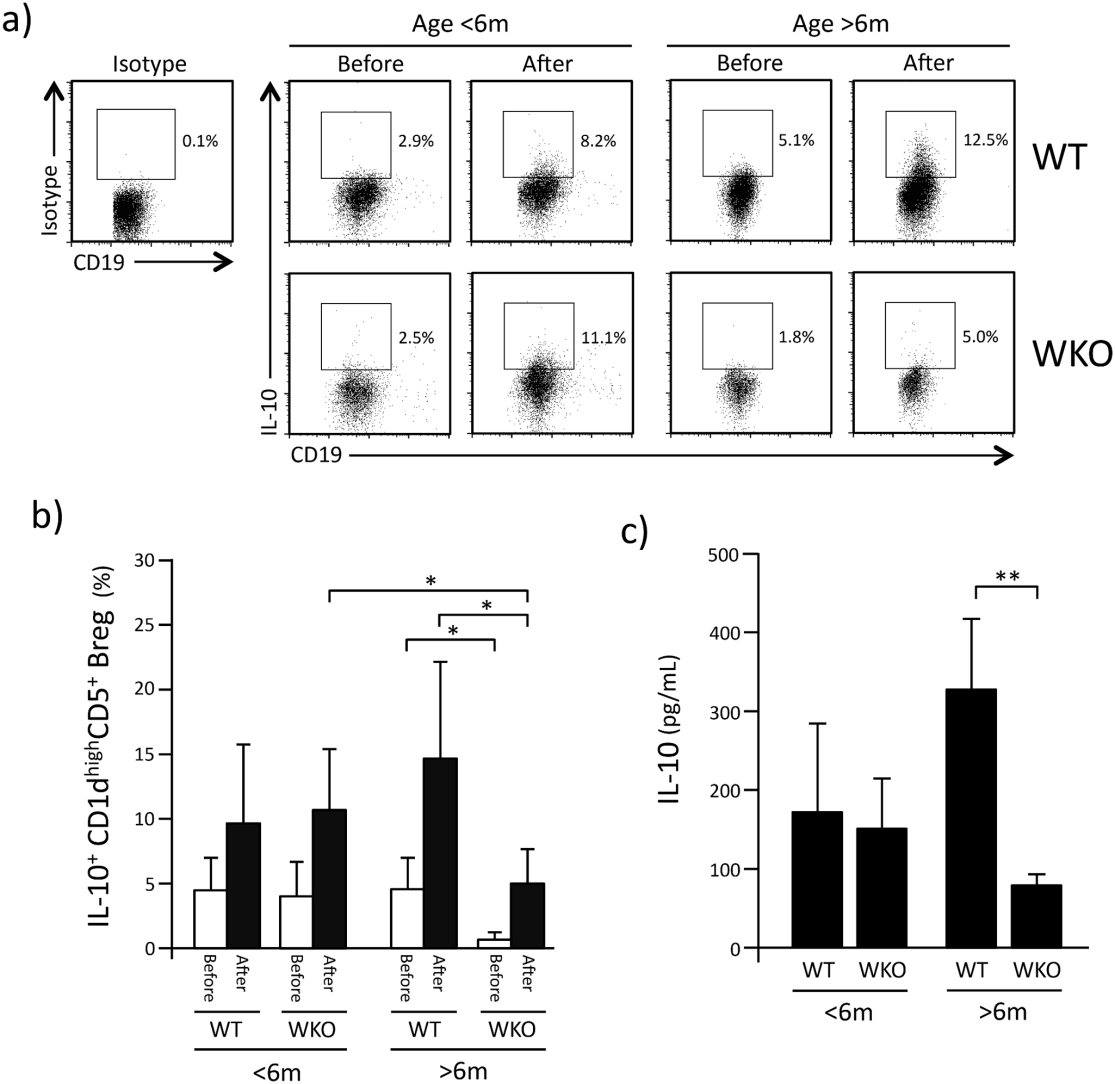
IL-10 production by WKO CD1d^high^CD5^+^ Breg. a: Representative flow-cytometry histograms showing IL-10-producing cells identified within CD1d^high^CD5^+^ Breg cells of younger (<6 months old) and older (>6 months old) mice before and 5 hrs after stimulation with LPS, PMA, and ionomycin. b: Percentage of IL-10-positive cells within CD1d^high^CD5^+^ Breg. White bars indicate the percentage (±SD) of IL-10-positive cells before stimulation (Before). Black bars indicate the percentage (±SD) IL-10 positive cells 5 hrs after stimulation with LPS, PMA and ionomycin (After). Each histogram represents data observed in 10 to 27 mice. c: IL-10 production after stimulation of CD1d^high^CD5^+^ Breg cells with LPS, PMA and ionomycin. Bar graphs indicate mean (±SD) of 3–5 experiments in which CD1d^high^CD5^+^ Breg cells were pooled from 6–10 mice were analyzed. *p<0.05, **p<0.002 (Student’s *t*-test).

We then assessed the proportion of IL-10-producing CD1d^high^CD5^+^ Breg cells within WT and WKO splenocytes in resting conditions (Before) and 5hrs after stimulation with LPI (After, Fig 2a). For both resting and stimulated splenocytes from younger animals, the proportion of IL-10-producing CD1d^high^CD5^+^ Breg cells did not differ between WT and WKO mice (Fig 2b). However, in WKO mice older than 6 months, the percentage of IL-10^+^ CD1d^high^CD5^+^ Breg cells both in resting and stimulated splenocytes was statistically lower than that detected in age-matched WT control mice. Moreover, the percentage of IL-10^+^ CD1d^high^CD5^+^ Breg cells in older WKO mice cells was also statistically lower compared to younger WKO mice (p<0.05)(Fig 2b).

Accordingly, while the concentration of IL-10 detected in the culture medium of stimulated CD1d^high^CD5^+^ Breg cells from younger WKO mice did not differ from WT control mice, IL-10 production by CD1d^high^CD5^+^ Breg cells from older WKO mice was statistically lower than that observed in age-matched WT CD1d^high^CD5^+^ Breg cells (Fig 2c, p<0.002).

**Fig 3:**
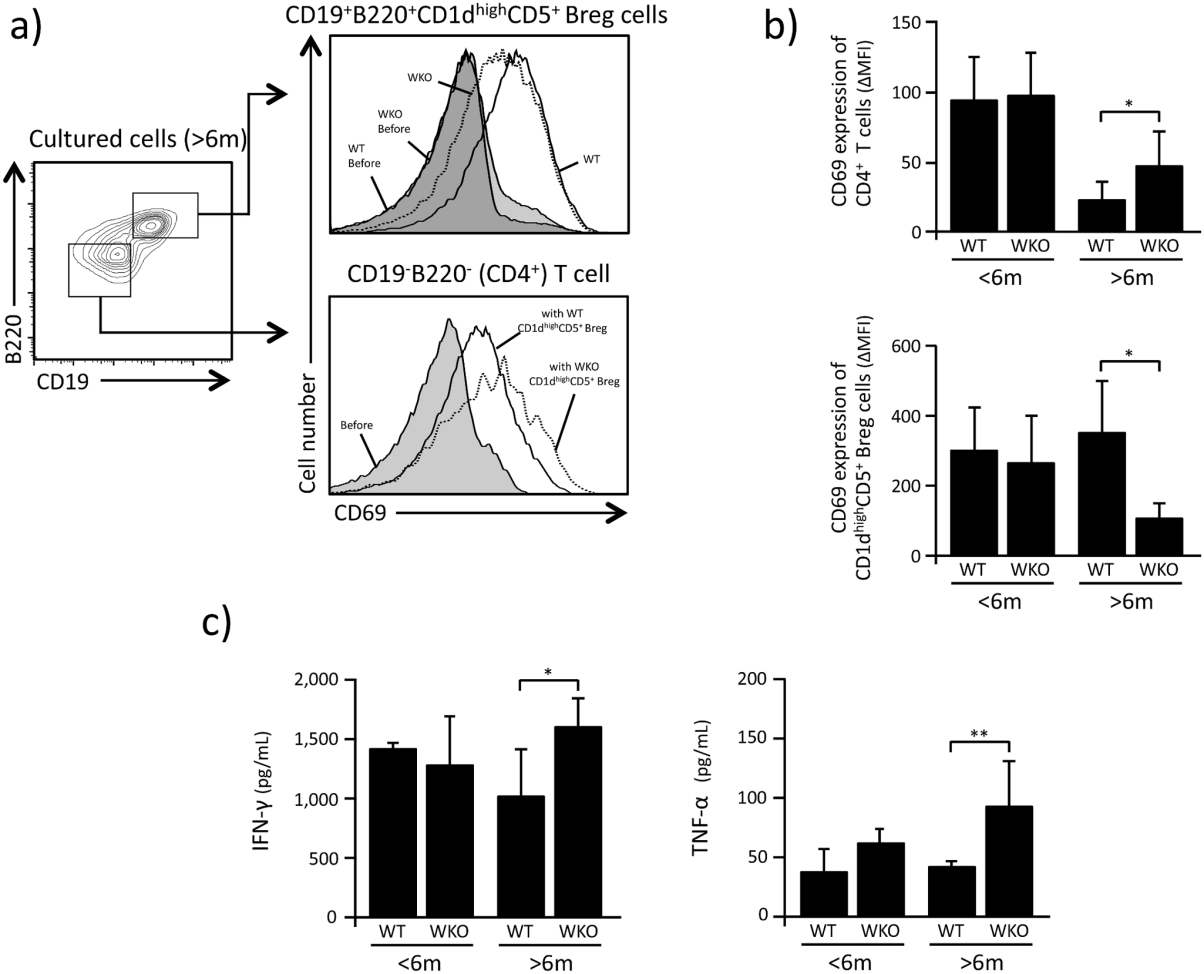
CD1d^high^CD5^+^ Breg suppression activity. a: Representative results showing the fluorescent intensity of CD69 expression on WT CD4^+^ T cells and CD1d^high^CD5^+^ Breg cells 72 hrs after co-culture with anti-CD3/CD28. b: Fluorescence intensity of CD69 expression on the surface of CD4^+^ T cells and CD1d^high^CD5^+^ Breg cells. Fluorescence intensity was expressed as delta mean fluorescence intensity (ΔMFI), which was calculated by subtracting the intensity of expression in unstimulated cells from that of cultured cell populations. Bar graphs indicate mean ± SD. *p<0.05 (Student *t*-test). c: Concentrations of cytokine (IFN- and TNF-) measured in the medium of CD1d^high^CD5^+^ Breg cells and CD4^+^ T cells co-cultured with LPS, PMA and ionomycin. Bar graphs indicate mean ±SD of 3–5 experiments in which CD1d^high^CD5^+^ Breg cells pooled from 6–10 mice were analyzed. *p<0.05, **p<0.02 (Student’s *t*-test).

CD1d^high^CD5^+^ Breg cells have been reported to suppress the activation of CD4^+^ T cells and affect their production of IFN- and TNF- (19). Based on the observations described above, we hypothesized that CD1d^high^CD5^+^ Breg cells from older WKO mice would show defective IL-10-mediated suppressing capabilities compared to CD1d^high^CD5^+^ Breg cells from older WT animals, as well as younger WKO mice. Indeed, in our co-culture experiments of CD4^+^ T cells and CD1d^high^CD5^+^ Breg cells, WKO CD1d^high^CD5^+^ Breg cells from older WKO mice showed lower suppression of CD69 expression on WT CD4^+^ T cells compared to CD1d^high^CD5^+^ Breg cells from age-matched WT (Fig 3a and 3b, p<0.05). We also noted that the CD69 expression levels on cultured CD1d^high^CD5^+^ Breg cells from older WKO mice was statistically lower than that of CD1d^high^CD5^+^ Breg cells from age-matched WT controls (p<0.05). Of note, no differences were observed between the effects of CD1d^high^CD5^+^ Breg cells from younger WKO and WT mice on the expression of CD69 on the surface of co-cultured CD4+ T cells (Fig 3a and 3b).

We also found that the concentration of IFN- and TNF- in the medium of co-cultures of WT CD4^+^ T and WKO CD1d^high^CD5^+^ Breg cells from older mice was statistically higher than that from co-cultures of control WT CD4^+^ T and WT CD1d^high^CD5^+^ Breg cells (IFN-: p<0.05, TNF-: p<0.02) (Fig 3c).

**Fig 4:**
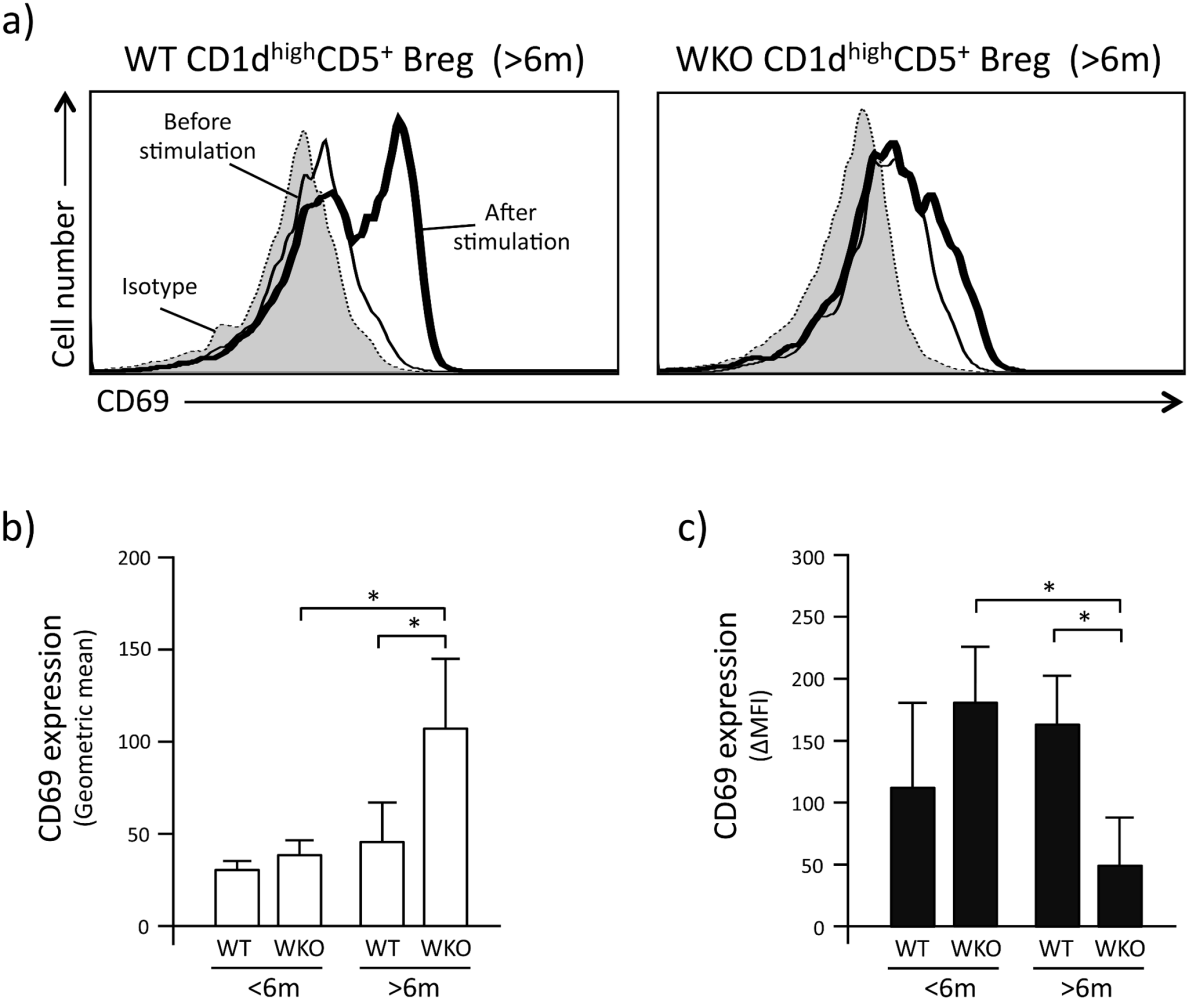
Activation status of CD1d^high^CD5^+^ Breg cells before/after LPI stimulation. a: Representative flow-cytometry histograms showing the expression of CD69 on CD1d^high^CD5^+^ Breg cells. Gray curves indicate isotype staining of the CD1d^high^CD5^+^ Breg cells. Thin and thick lines indicate CD69 expression before and after LPI stimulation, respectively. b: CD69 expression intensity on CD1d^high^CD5^+^ Breg cells before LPI stimulation expressed as geometric mean. c: Change in intensity of CD69 expression on CD1d^high^CD5^+^ Breg cells after LPI stimulation. Data are expressed as ΔMFI, calculated as in Fig 3b. Data are mean and SD of samples obtained from 6–10 mice in each assays and repeated 3–5 times. *p<0.001 (Student’s *t*-test).

To investigate whether the lower production of IL-10 by CD1d^high^CD5^+^ Breg cells from older WKO mice was due to reduced effects of LPI stimulation, we assessed the expression levels of CD69 on the surface of CD1d^high^CD5^+^ Breg cells before and after LPI stimulation (Fig 4a).

Interestingly, we found that the CD69 expression levels on CD1d^high^CD5^+^ Breg cells from older WKO mice before LPI stimulation was statistically higher than that of CD1d^high^CD5^+^ Breg cells from age-matched WT controls and younger WKO mice (Fig 4b, p<0.001). In contrast, after LPI stimulation, the CD69 expression levels of CD1d^high^CD5^+^ Breg cells from older WKO mice were statistically lower than that of CD1d^high^CD5^+^ Breg cells from age-matched WT and younger WKO controls (Fig 4c, p<0.05 and 0.001, respectively).

Our observations show that age plays a significant role in shaping the CD19^+^CD1d^high^CD5^+^ B cell compartment of WKO mice. When analyzed at >6 months of age, WKO mice present significantly lower percentages of IL-10 producing CD1d^high^CD5^+^ Breg cells compared to both WT control and young WKO mice. This condition results in reduced suppression of CD4^+^ T-cell activation and decreased inhibition of IFN- and TNF- production. Altogether, older WKO mice show significantly impaired regulatory B cell function that may contribute to the onset of the known autoimmune complications observed in this animal model and possibly to the age-dependent exacerbation of the autoimmune phenotype that we have previously described [15, 20].

Further studies are needed to uncover the mechanisms leading to these findings. As shown by Bouma et al. [9], WKO mice of less than 6 months of age show a more severe experimental arthritis compared to control mice that can be ameliorated by adoptive transfer of Breg cells. By comparing equal numbers of WT and WKO CD1d^high^CD5^+^ Breg cells, we demonstrate here that WKO CD1d^high^CD5^+^ Breg cells from young WKO mice maintain the ability of secreting normal amounts of IL-10. The increased severity of the autoimmune arthritis observed in these mice, therefore, can simply be due to the reduction of Breg cell frequency observed by Bouma et al. and the lower absolute numbers of CD1d^high^CD5^+^ Breg cells identified by us in WKO mice. Interestingly, we demonstrated that IL-10 secretion of WKO CD1d^high^CD5^+^ Breg cells from older mice becomes significantly defective. Similar to the observations we previously made in WKO nTreg cells that have significantly reduced ability of granzyme B degranulation [8], the defective secretion of IL-10 by WKO CD1d^high^CD5^+^ Breg cells may be attributable to defective cytokine trafficking and release due to aberrant cytoskeletal reorganization in the absence of WASp expression. However, the absence of similar findings in CD1d^high^CD5^+^ Breg cells from younger WKO mice makes this explanation somewhat less compelling. On the other hand, our findings that WKO CD1d^high^CD5^+^ Breg cells from older mice present with significantly higher levels of CD69 expression in basal conditions suggest that the functional impairment of CD1d^high^CD5^+^ Breg cells from older WKO mice may be at least in part due to abnormal activation, possibly translating in defective responses to further stimulation. The reasons underlying the increased levels of cellular activation in CD1d^high^CD5^+^ Breg cells from older WKO mice are at present unclear.

## Conclusions

Our observations uncovered additional characteristics of the defective compartment of regulatory B cells that develops in the absence of WASp expression and provides possible insights into the development of autoimmunity with increasing age in patients affected with WAS.

## Supporting Information

S1 Fig Gating strategy for evaluation of CD1^high^CD5^+^ Breg cells. (PDF)
