# Supplementary figures and images for: Age-Dependent Defects of Regulatory B Cells in Wiskott-Aldrich Syndrome Gene Knockout Mice
Source: PLoS One. 2015 Oct 8;10(10):e0139729. doi: 10.1371/journal.pone.0139729 (PMC4598155; doi:10.1371/journal.pone.0139729)

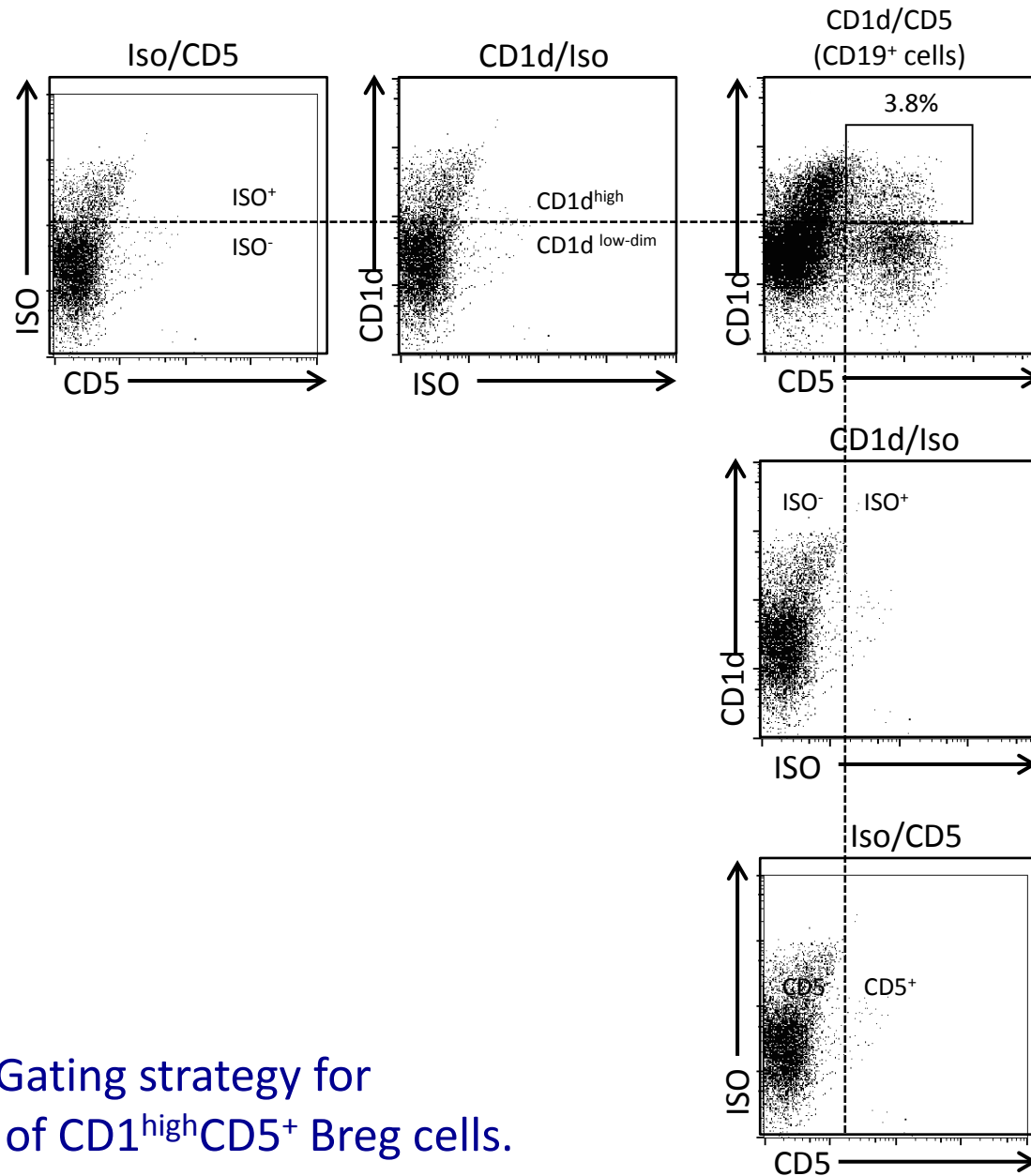

**S1 Figure:** Gating strategy for evaluation of CD1<sup>high</sup>CD5<sup>+</sup> Breg cells.

Supplement: S1 Fig — (PDF) [file pone.0139729.s001.pdf]
